# Supplementary material for: Monitoring and responding to emerging infectious diseases in a university setting: A case study using COVID-19
Source: PLoS One. 2023 May 17;18(5):e0280979. doi: 10.1371/journal.pone.0280979 (PMC10191342; doi:10.1371/journal.pone.0280979)
Supplement: S1 Appendix — (DOCX) [file pone.0280979.s001.docx]

**S1 Appendix. Derivation of model parameters and detailed targeted sampling description.**

**Estimating the Rate of Imported Infections and COVID-19 Prevalence.**

In the modified version of Covasim used in our study, the number of imported infections on each day is a Poisson random variable whose rate parameter differs between the residential and non-residential sub-populations. Under the assumption that the community surrounding the university is well mixed, the rate parameter for sub-population *i* (*λ_i_*) was assumed to be:

$$\lambda_{i}=n_{i}c_{i}p\beta$$

(1)

Here, *n_i_* is the size of sub-population *i*, *c_i_* is the expected number of contacts between one student in sub-population *i* and non-student members of the community, *p* is the COVID prevalence in the university's surrounding community, and β is the base transmission probability. Whereas *n_i_* and β were already parameters within the model, *c_i_* and *p* required additional specification.

To determine reasonable values for *c_i_*, we established the average number of contacts that each student will have in a day, excluding roommates, based on empirical estimates in Bharti et al. (1), then allocated these contacts to the in-simulation contact pools and to out-of-system community members. For residential students, contacts were predominantly allocated to the in-system pools; only one contact per day or one contact every other day was allocated to out-of-system individuals (Table 2 in main text). In contrast, non-residential students used the same number of community contacts as residential students, but all remaining contacts were allocated to out-of-system individuals. Here, we assumed individuals living outside of university-owned properties were more likely to interact with non-students.

Within this study, the area surrounding the university was defined as the entire state of Connecticut. Then, case incidence data led to rough estimates for *p*. All case incidence data came from COVID Act Now and were smoothed as seven-day averages (2). To estimate *p*, we decomposed the number of infectious individuals on a given day (*N_it_*) into the number of symptomatic individuals (*N_st_*), the number of asymptomatic individuals (*N_at_*), and the number of pre-symptomatic individuals (*N_pt_*). We assumed there was a fixed probability that a symptomatic resident of Connecticut led to a reported case on any given day (*p_d_*) and that only symptomatic individuals contribute to the reported incidence rate. Under these assumptions, the number of days between symptom onset and detection is a geometric random variable, but in some cases, the symptomatic individual will recover before being detected. Therefore, the probability that a symptomatic infection will eventually lead to a reported case (*p_D|S_*) would be the following:

$$p_{D|S}=\sum_{i=1}^{T} p_{d}\left( 1-p_{d} \right)^{i-1}$$

(2)

Here, *T* is the duration between symptom onset and recovery. Furthermore, define the reporting rate (*ρ*) as the probability that a randomly selected infection, be it symptomatic or asymptomatic, will lead to a reported case. Under this definition, *ρ* = *p_D|S_p_S_*, where *p_S_* is the probability that an infection will be symptomatic.

Based on Khalili *et al.* (3), we set *T* to 18 days. Furthermore, CDC seroprevalence studies in Connecticut estimated that between 12.61% and 22.77% of all infections led to a reported case (4); therefore, we set *ρ* to 0.20. Finally, we set *p_S_* to 0.6 based on Oran and Topol (5). Based on these estimates and Equation 2, we used numerical optimization algorithms in R ver. 4.2 to solve for *p_d_* under the constraint that the value must be between 0 and 1 (6) (S2 Supporting Information). The estimated value was 0.022274.

The previous assumptions further imply that the total number of reported cases on a given day is a binomial random variable where the number of trials is *N_st_* and the probability of success is *p_d_*. Since the binomial distribution is unimodal, we estimated *N_st_* as the integer value that places the distribution’s mode at the observed number of reported cases on day *t* (*n_cases,t_*) under a strategy akin to maximum estimation. The mode of this binomial distribution is $\left\lfloor\left( N_{st}+1 \right)p_{d} \right\rfloor$, where $\left\lfloor\ldots\right\rfloor$ is the floor function, so the estimate was roughly:

$$\hat{N}_{st}=\frac{n_{cases,t}}{p_{d}}-1$$

(3)

The remaining estimates for *N_at_* and *N_pt_* followed from $\hat{N}_{st}$. Since:

$$p_{s}\approx\frac{N_{st}}{N_{st}+N_{at}}$$

(4)

An approximate estimate for *N_at_* is:

$$\hat{N}_{at}=N_{st}\frac{1-p_{s}}{p_{s}}$$

(5)

Finally:

$$\hat{N}_{pt}=\sum_{j=1}^{d} \left( \hat{N}_{at+j}+\hat{N}_{st+j} \right)$$

(6)

Where *d* is the duration between when an individual becomes infectious and when the individual develops symptoms, assuming a symptomatic infection. Based on Tindale *et al.* (7)*,* we set *d* to three days. Our method for estimating *N_pt_* represents an upper limit, though, as it assumes that symptomatic individuals or individuals that could be established as asymptomatic on day *i* do not overlap with those same groups on day (*i* + 1). Finally, to convert these estimates to a final prevalence for the state of Connecticut, the estimates were summed and divided by the estimated population of Connecticut according to the US Census Bureau’s July 1, 2019 estimate (8). R code for implementing these calculations is available in S2 Supporting Information.

**Derivation of a Relationship Between R_0_ and the Probability of Transmission.**

Covasim does not explicitly use the basic reproductive number (*R*_0_) as a model parameter. The closest analog is the base probability of transmission (*β*). Yet, because *R*_0_ is a prominent parameter in public health guidelines, there is a utility to linking *β* to an *a priori* value for *R*_0_ in a manner that is consistent with Covasim’s assumptions and its default values for epidemiological parameters. Assume that there is a well-mixed population of susceptible individuals, that this population is arbitrarily large, and that disease transmission occurs in a manner identical to its depiction in Covasim. Define *R*_0_ as the expected number of new infections in this population that directly result from introducing a single infectious individual. Since the population is arbitrarily large and well-mixed, the probability that the infectious individual will contact the same susceptible individual twice is approximately zero. Therefore, under Covasim’s assumptions on disease transmission, the number of new infections depends on the total number of contacts the infectious individual makes before recovery and on the probability of transmission. Further, every contact is a Bernoulli trial that ends in either transmission or no transmission. Therefore, *R*_0_ is the sum of the expected probability of transmission for each contact.

The probability of transmission for each contact is the product of *β* and two scaling factors. The first factor represents the relative susceptibility of the contacted individual (*s_j_*), which is age-dependent (Table A) (9). Therefore, the expected susceptibility is the sum of each age group’s susceptibility weighted by the proportion of the population that belongs to that age group. To establish this expected value, we used the age distribution of the entire US population rather than the population of the university, as this made our definition of *R*_0_ more consistent with that of the broader literature (Table A).

**Table A.** The susceptibility constants associated with each age group in Covasim’s defaults, as well as the proportion of the US population that belongs to each age group (10).

| **Age Group** | **Susceptibility Constant** | **Proportion of the US Population** |
| --- | --- | --- |
| 0-9 | 0.34 | 0.121 |
| 10-19 | 0.67 | 0.131 |
| 20-59 | 1 | 0.525 |
| 60-69 | 1.24 | 0.1095* |
| +70 | 1.47 | 0.1135* |

* These values slightly differ from the proportions in (10). There, the population proportion in the 60-69 age group is 0.115, and the population proportion in the over 70 age group is 0.108. The values have been left as-is for consistency with the model used in fall 2020. Correcting this difference minorly changes the relationship to $\beta\approx\frac{R_{0}}{{8.083054n}_{c}}$ and does alter β by more than 6x10^-5^.

The second scaling factor represents the relative transmissibility of the infectious individual at the time of contact (*r_it_*), which accounts for the infectious individual’s viral load. Covasim’s defaults assume that, in most cases, the transmissibility scaling factor is two during the first 30% of the infectious period and one during the final 70%. Assuming this generalization holds for the infectious individual, *R*_0_ can be expressed as the sum of the expected number of new infections during the first 30% of the infectious period and the expected number during the last 70%, which simplifies subsequent calculations. The duration of a simulated infectious period is a log normal random variable that depends on health outcomes, such as whether an individual is asymptomatic or symptomatic, whether the individual requires hospitalization, and whether the individual requires admission to an intensive-care unit. We assumed the infectious period was nine days long, which is Covasim’s default expected duration for an asymptomatic infection or a symptomatic infection that does not require hospitalization. Since the model uses a one-day timestep, this means that the period of elevated transmissibility lasts two days whereas the period of lower transmissibility lasts seven days. In addition to viral load, *r_it_* is further scaled by a random constant that is unique to that individual (*c_i_*), which accounts for idiosyncratic factors (*e.g.,* comorbid conditions, genetic factors, behavioral factors). Since $100c_{i} \sim NegBinom\left( 0.45,\frac{0.45}{100.45} \right)$, E[*c_i_*]=1.

Put together:

$$R_{0}=E\left[ 2\left( n_{c,h}c_{i}s_{j}\beta\right)+n_{c,l}c_{i}s_{j}\beta\right]=(2E\left[ n_{c,h} \right]+E\left[ n_{c,l} \right])E\left[ c_{i} \right]E\left[ s_{j} \right]\beta$$

Here, *n_c,h_* is the number of contacts the infectious individual has during the first 30% of the infectious period, *n_c,l_* is the number of contacts the infectious individual has during the final 70% of the infectious period, and all other parameters are as defined above. Notice that although *n_c,h_*, *n_c,l_*, *c_i_*, and *s_j_* are all random variables, they are all mutually independent, so the second equality holds. Recalling that the model assumes that the number of contacts an individual has in one day is a Poisson random variable, E[*n_c,h_*] and E[*n_c,l_*] are the expected number of contacts in one day times the duration of each parameter’s associated period. Adding the assumption that the infectious period is nine days, the expression can be rearranged to provide an approximate relationship between *R_0_* and *β* when the expected number of contacts (*n_c_*) is known:

$$\beta\approx\frac{R_{0}}{{8.766238n}_{c}}$$

R code for implementing these calculations is available in S2 Supporting Information.

**Detailed Sampling Strategy.**

All students had to provide confirmation of a negative COVID test in late August prior to the start of the fall semester and were tested again on campus at the beginning of the semester. After this, a targeted sampling plan for weekly COVID testing began.

***Residential Undergraduate Students***

We applied a strict stratified and cluster sampling method to students in residential dormitory buildings. Dormitories at Quinnipiac University have varied floor plans. Some dormitories contain typical student dorm rooms along a hallway, while other buildings have suites in which students share a living area and bathrooms but have separate bedrooms. Since students in the same suite lived in such close proximity, though, suites were treated as if they were a single dorm room. Similarly, the university owns multiroom houses that serve as student residences, but because students in the same house frequently contacted each other, QU-owned houses were treated comparably to a single dorm room. Occupancy per room varies largely by building, suite, or house. Starting in the fall of 2020, the university allowed a maximum of two students per dorm room. To guarantee at least one student from a close-contact living space was selected, we defined building floors (*i.e.*, those containing dorm rooms) and houses as strata in stratified sampling. We utilized a proportionate sampling method to determine the number of students being selected from each stratum and then used cluster sampling to draw students from dorm rooms, suites, and houses.

***Non-Residential Undergraduate Students***

About 40% of undergraduate students commuted to school. They either lived with relatives or rented an apartment or house in the local community. Since they had greater exposure to the community, the plan was to sample 25% of non-residential undergraduates each week. Based on student records, we could determine the number of students who shared an address. Students who shared an address with no more than one other student were entered in a pool for simple random sampling (SRS). Students who shared the same address with two or more students (*i.e.,* the residence contained at least three students), including those living in the same apartment building as other students, were kept in a separate pool from which at least one student was selected weekly to be tested.

***Graduates and Student Athletes***

We assumed that graduate students, even those living off campus, would be more responsible and exhibit more precautious behaviors than undergraduates. Thus, we used SRS with a smaller sampling rate of 15%. However, graduate students who shared the same residence with undergraduate students or who lived in university-owned properties were placed in the undergraduate student pool and sampled in the same way as undergraduate students. Finally, we sampled 80% of student athletes weekly to meet the requirements set by the Metro Atlantic Athletic Conference of the National Collegiate Athletic Association. Student athletes were stratified by team.

**Table B**. Weekly proportion of students sampled and case incidence for each week.

|  |  | **Sampling Percentages** | | | |
| --- | --- | --- | --- | --- | --- |
| **Week Ending Day** | **Case Incidence** | **Res Undergrad** | **Non-res Undergrad** | **Graduate** | **Athletes** |
| Pre-arrival | - | 100% | 100% | 100% | 100% |
| 08/30/2020 | - | 100% | 100% | 100% | 100% |
| 09/06/2020 | 2 | 100% | 100% | 100% | 100% |
| 09/13/2020 | 0 | 15% | 25% | 15% | 80% |
| 09/20/2020 | 1 | 15% | 25% | 15% | 80% |
| 09/27/2020 | 0 | 15% | 25% | 15% | 80% |
| 10/04/2020 | 3 | 15% | 25% | 15% | 80% |
| 10/11/2020 | 20 | 15% | 35% | 15% | 80% |
| 10/18/2020 | 7 | 15% | 35% | 15% | 80% |
| 10/25/2020 | 18 | 15% | 35% | 15% | 80% |
| 11/01/2020 | 48 | 15% | 60% | 40% | 80% |
| 11/08/2020 | 296 | 15% | 60% | 40% | 80% |
| 11/15/2020 | 151 | 100% | 100% | 100% | 100% |
| 11/22/2020 | 67 | 25% | 25% | 15% | 80% |

Res Undergrad = Residential undergraduate students, Non-res Undergrad = Non-residential undergraduate students, Graduate = Non-residential graduate students, Athletes = Student athletes

**References**

1. Bharti N, Exten C, Fulton V, Oliver-Veronesi R. Lessons from managing a campus mumps outbreak using test, trace, and isolate efforts. Am J Infect Control. 2021 June;49(6):849–51.

2. Covid Act Now. Covid Act Now API; 2022 [cited 2022 Jul 21]. Database: Covid Act Now. Available from: https://apidocs.covidactnow.org/

3. Khalili M, Karamouzian M, Nasiri N, Javadi S, Mirzazadeh A, Sharifi H. Epidemiological characteristics of COVID-19: a systematic review and meta-analysis. Epidemiol Infect. 2020;148(e130):1–17.

4. Havers FP, Reed C, Lim T, Montgomery JM, Klena JD, Hall AJ, et al. Seroprevalence of Antibodies to SARS-CoV-2 in 10 Sites in the United States, March 23-May 12, 2020. JAMA Intern Med. 2020 July 21;180(12):1576–86.

5. Oran DP, Topol EJ. Prevalence of asymptomatic SARS-CoV-2 infection: a narrative review. Ann Intern Med. 2020;173(5):362–7.

6. R Core Team. R: A Language and Environment for Statistical Computing. Version 4.2.1 [software]. 2022 [cited 2023 Jan 18]. Available from: https://www.r-project.org/

7. Tindale LC, Coombe M, Stockdale JE, Garlock ES, Lau WYV, Saraswat M, et al. Transmission interval estimates suggest pre-symptomatic spread of COVID-19. medRxiv: 2020.03.03.20029983 [Preprint]. 2020 Feb [cited 2023 Jan 9]: [30 p.]. Available from: https://www.medrxiv.org/content/10.1101/2020.03.03.20029983v1.full.pdf

8. U.S. Census Bureau. 2019: PEP Population Estimates; 2019 [cited 2023 Jan 9]. Database: data.census.gov [Internet]. Available from: https://data.census.gov/cedsci/table?g=0400000US09&tid=PEPPOP2019.PEPANNRES

9. Kerr CC, Stuart RM, Mistry D, Abeysuriya RG, Rosenfeld K, Hart G, et al. Covasim : an agent-based model of COVID-19 dynamics and interventions. PLoS Comput Biol. 2021;17(7):e1009149.

10. U.S. Census Bureau. 2018: ACS 1-Year Estimates Subject Tables; 2018 [cited 2022 Jul 28]. Database: United States Census Bureau [Internet]. Available from: https://data.census.gov/table?q=age+distribution+2018&tid=ACSST1Y2018.S0101
