# Supplementary material for: Monitoring and responding to emerging infectious diseases in a university setting: A case study using COVID-19
Source: PLoS One. 2023 May 17;18(5):e0280979. doi: 10.1371/journal.pone.0280979 (PMC10191342; doi:10.1371/journal.pone.0280979)
Supplement: Attachment — Submitted filename: SodaChenFeinnHill_PLOSOne_ResponseLetter.pdf [file pone.0280979.s003.pdf]

Quinnipiac
College of Arts & Sciences

Quinnipiac University
275 Mount Carmel Avenue
Hamden, CT 06518
Kenneth.Soda@quinnipiac.edu

Barbara T. Rumain, PhD
Academic Editor
*PLOS ONE*

April 11, 2023

Dear Dr. Rumain:

My co-authors and I were glad to receive your invitation to revise and resubmit our paper, "Monitoring and responding to emerging infectious diseases in a university setting: A case study using COVID-19" (Manuscript PONE-D-23-00956), for publication in *PLOS ONE* on March 14, 2023. We thank you and our reviewers for the comments that you provided to strengthen our work. Now that we have incorporated this feedback into our manuscript, we are writing to resubmit our manuscript for your further consideration.

We have improved our manuscript based on every comment that the reviewers provided to us. In addition, while verifying that our reference list is complete and correct, we found a minor discrepancy between a value provided in a referenced paper and a value quoted in our S1 Appendix. This too has been corrected. Our manuscript with tracked changes highlights these improvements, and per your instructions, we have also provided a clean copy without tracked changes. The corrections to our supplementary appendix are only provided via a clean copy, but a version with tracked changes is also available if you or our reviewers would like to review it. We directly address each comment in the tables below and summarize what modifications have been made since our first submission.

Responses to Reviewer #1

| **Comment and Response** | **Relevant Page and Line in Manuscript with Tracked Changes** |
| --- | --- |
| Comment: The authors present a modified agent-based covid-19 model based on Covasim. The modifications are explained in detail and are based on disease mitigation strategies implemented by Quinnipiac University in the Fall 2020 semester. The results of the model are then compared to observed data from that semester. Suggestions for future infectious diseases at institutes of higher educations are also provided.<br><br>Response: We believe that this is an accurate summary of our work and goals. | |
| Comment: The model and methodology for analyzing it appear sound and thorough.<br><br>Response: We thank the reviewer for this favorable assessment. | |

| | |
|---|---|
| Comment: My main questions are regarding the main four scenarios:<br><br>Can the authors provide the rationale for choosing the two specific R_0 values used in the study? Did these come from known values at the time or were they completely hypothetical?<br><br>Response: Our main four scenarios used two values for $R_0$, 1.5 and 2.5. Both these values were informed and later validated by the literature. We selected the $R_0$ value of 2.5 because the (US) Centers for Disease Control and Prevention's COVID-19 Pandemic Planning Scenarios, as published on May 20, 2020, listed 2.5 as their best estimate for $R_0$. In addition, Li *et al.* (2020) estimated a similar value for $R_0$ (2.2) using case data in Wuhan, China from between December 10, 2019, and January 4, 2020.<br><br>Our model did not directly use $R_0$ as a model parameter. Instead, we used $R_0$ to establish reasonable values for the probability that a social contact between an infectious individual and a susceptible individual would lead to disease transmission. However, we also believed that public health interventions, such as social distancing and masking, would also lower the probability of transmission. To account for these effects, we also incorporated scenarios where $R_0$ was 1.5, partially in convention with one of the effective reproductive numbers used in Paltiel, Zheng, and Walensky (2020). Later, Pitzer *et al.*(2021) retroactively validated this choice through their estimates for the effective reproductive rate in the United States during the summer of 2020.<br><br>The rationale provided above, along with the relevant citations, have now been added to the manuscript. | pg. 11, Lines 230-238 |
| Comment: Also, the authors cite Bharti et al. for the average contact rates of 8 and 10, which was published in 2021. However, the authors state that their model was created in summer 2020 to help plan for fall 2020. Was the preprint mentioned in the Acknowledgments available to the authors for their initial use of the model? This point could be made clear.<br><br>Response: The reviewer is correct that Dr. Bharti provided us with a preprint of her and her colleagues' paper before it was published, and this preprint informed our work. We now explicitly state this in the main text. | pg. 11, Lines 238-240 |

Responses to Reviewer 2

| **Comment and Response** | **Relevant Page and Line in Manuscript with Tracked Changes** |
|---|---|
| Comment: This manuscript provides insight into one university's experience with COVID-19 modeling and disease mitigation. It provides detailed information related to disease modeling strategies and observed disease transmission that would be useful to other IHEs for future use of Disease X modeling. Additionally, they present practical considerations related to university prevention strategies. Comparisons with other IHE experiences highlight similar strategies/outcomes as well as differing experiences that call for further investigation.<br><br>Response: We believe that this is an accurate summary of our work. | |
| Comment: To provide additional clarity for the reader in understanding the figures, the following edits are recommended: Given that Figure 1 includes predicted and observed values, include "predicted" in the manuscript description and the figure labeling.<br><br>Response: We now explicitly mention that Figure 1 contains both predicted and observed values, both within the in-text description and in the figure title, and are more careful to differentiate predicted results from observed results. The figure legend has also been updated to emphasize that the median predictions and confidence intervals were based on the same 1,000 replicate simulations. | pg. 16, Lines 343-347<br>pg. 16, Lines 351-352<br>pg. 16, Line 354 |
| Comment: With regard to Figure 3, the manuscript text does state that the figure shows predicted values; adding this to the figure label would provide further clarity.<br><br>Response: Both the figure title and figure legend now explicitly state that Figure 3 shows predicted values. Just as in Figure 1, we also updated the legend to emphasize that the median predictions and confidence intervals were based on the same 1,000 replicate simulations. | pg. 19, Lines 407-408<br>pg. 19, Lines 410 |
| Comment: Label y-axis for figures 1 and 3.<br><br>Response: Figures 1 and 3 now have labelled y-axes. | Fig1.tif<br>Fig3.tif |

Additional Modifications and Formatting Changes

| Comment and Updates |
|---|
| Comment: When submitting your revision, we need you to address these additional requirements.<br><br>1. Please ensure that your manuscript meets PLOS ONE's style requirements, including those for file naming. The PLOS ONE style templates can be found at |

https://journals.plos.org/plosone/s/file?id=wjVg/PLOSOne_formatting_sample_main_body.pdf and

https://journals.plos.org/plosone/s/file?id=ba62/PLOSOne_formatting_sample_title_authors_affiliations.pdf

Response: We have reviewed PLOS ONE's style requirements and have updated the manuscripts formatting accordingly. These updates include:

- Adding my initials after my email address on the title page (pg. 1, line 15).
- Using Microsoft Word's Heading styles for section and subsection titles (pg. 1, lines 17, 40; pg. 6, lines 121-122; pg. 7, line 139; pg. 8, lines 160, 168, 178; pg. 10, line 213; pg. 12, line 263; pg. 14, line 288; pg. 15, lines 316, 322-323; pg. 16, line 342; pg. 17, line 379; pg. 18, line 394; pg. 21, lines 452-453; pg. 22, line 484; pg. 24, line 528; pg. 26, line 583; pg. 28, line 641; pg. 29, line 662; pg. 30, line 679; pg. 32, line 736; pg. 33, lines 742, 751; pg. 40, line 929).
- Correcting an instance where Insert→Equation was used to provide a single variable (pg. 8, line 177).
- Bolding table titles (pg. 11, line 247; pg. 12, lines 249-250).
- Changing all references to the S2 Additional Materials to references to the S2 Supporting Information so that the in-text references match the corresponding file's name (pg. 12, line 262; pg. 15, line 315). Similarly, we have changed the corresponding file's name from S2_SupportingInformation.zip to S2_Supporting_Information.zip for greater consistency with its in-text references.
- Running each figure through PACE to ensure that every figure adheres to the necessary figure requirements.
- Removing the Author Contributions section from the manuscript. Based on the style template, we believe that this information was not meant to be included in the manuscript itself, only in the submission forms. If we are mistaken, we will be happy to return this section (pg. 33, lines 742-750).

Comment: 2. Please review your reference list to ensure that it is complete and correct. If you have cited papers that have been retracted, please include the rationale for doing so in the manuscript text, or remove these references and replace them with relevant current references. Any changes to the reference list should be mentioned in the rebuttal letter that accompanies your revised manuscript. If you need to cite a retracted article, indicate the article's retracted status in the References list and also include a citation and full reference for the retraction notice.

Response: We reviewed our reference list, both in the main text and the S1 Appendix, and have reformatted any references that deviated from the Vancouver citation style. In this process, we discovered that the published version of Bharti *et al.* (2021) (Manuscript Reference 30; S1 Appendix Reference 1) had a slightly different title from that of the preprint. This has now been corrected. The same observation was made of Havers *et al.* (2020) in the S1 appendix (S1 Appendix Reference 4), and this citation was similarly corrected.

To address a comment from Reviewer 1, we have added the following references:

- National Center for Immunization and Respiratory Diseases (NCIRD), Division of Viral Diseases. COVID-19 Pandemic Planning Scenarios - May 20, 2020 [Internet]. Atlanta (GA): Centers for Disease Control and Prevention; 2020 [cited 2020 May 25].

Available from: https://www.cdc.gov/coronavirus/2019-ncov/hcp/planning-scenarios-archive/planning-scenarios-2020-05-20.pdf

- Li Q, Guan X, Wu P, Wang X, Zhou L, Tong Y, et al. Early transmission dynamics in Wuhan, China, of novel coronavirus–infected pneumonia. N Engl J Med. 2020 March 26;382(13):1199–207.
- Pitzer VE, Chitwood M, Havumaki J, Menzies NA, Perniciaro S, Warren JL, et al. The impact of changes in diagnostic testing practices on estimates of COVID-19 transmission in the United States. Am J Epidemiol. 2021;190(9):1908–17.

In addition, we discovered that the reference:

- Cohen JA, Stuart RM, Rosenfeld K, Lyons H, White M, Kerr CC, et al. Quantifying the role of naturally- and vaccine-derived neutralizing antibodies as a correlate of protection against COVID-19 variants. medRxiv: 2021.05.31.21258018 [Preprint]. 2021 [cited 2023 Jan 8]. Available from: https://www.medrxiv.org/content/10.1101/2021.05.31.21258018v2%0Ahttps://www.medrxiv.org/content/10.1101/2021.05.31.21258018v2.abstract

Should have been:

- Cohen JA, Stuart RM, Núñez RC, Wagner B, Chang S, Rosenfeld K, et al. Mechanistic modeling of SARS-CoV-2 immune memory, variants, and vaccines. medRxiv: 2021.05.31.21258018 [Preprint]. 2021 [posted 2021 June 1; cited 2023 Jan 18]: [19 p.]. Available from: https://www.medrxiv.org/content/10.1101/2021.05.31.21258018v2

Similarly, in the S1 Appendix, the reference:

- U.S. Census Bureau. 2020: DEC Redistricting Data (PL 94-171); 2021 [cited 2022 Jul 28]. Database: census.gov [Internet]. Available from: https://www.census.gov/quickfacts/newhavencountyconnecticut

Should have been:

- U.S. Census Bureau. 2018: ACS 1-Year Estimates Subject Tables; 2018 [cited 2022 Jul 28]. Database: United States Census Bureau [Internet]. Available from: https://data.census.gov/table?q=age+distribution+2018&tid=ACSST1Y2018.S0101

To the best of our knowledge, we have not cited any retracted articles. Virtanen *et al.* (2020) does have a correction, but the corrected paper appears to have the same citation as the original.

Additional Modifications Made While Proofreading and Double-Checking Citations

In addition to the changes mentioned above, we have made the following additional modifications:

- The phrase, "Relative to targeted sampling, complete sampling had 32.05 fewer infections at the under boundary under 8 contacts/day…", has been corrected to say, "Relative to targeted sampling, complete sampling had 32.05 fewer infections at the upper boundary under 8 contacts/day…" (pg. 19, line 403).
- We previously stated that Cas *et al.* (2021) discussed Furman University's COVID-19 response. Although this is implied within the paper, the authors only state that they are describing the response of a college in South Carolina. We have therefore removed Furman University's name to follow this convention (pg. 27, line 616; pg. 32, line 724).
- We have updated our description of Muller and Muller's (2021) results on pg. 31, Lines 696-697 to more accurately reflect the authors' findings on the effectiveness of

contact tracing relative to surveillance testing with complete or nearly complete sampling.

The description previously read, "This intervention has a long and proven track record in public health policy, translates well to novel diseases, and was found to have a comparable effectiveness to surveillance testing with complete sampling in other modeling studies of COVID-19 (41)."

The description now reads, "This intervention has a long and proven track record in public health policy, translates well to novel diseases, and was found to have a comparable effectiveness to surveillance testing with complete or nearly complete sampling in other modeling studies of COVID-19, at least under certain measures (44)."

- In our S1 Appendix, we previously stated that a CDC seroprevalence study in Connecticut estimated that between 12.82% and 23.26% of all infections led to a reported case. A second review of the paper (Havers *et al.* 2020) found that it was actually between 12.61% and 22.77% of all infections. This statement has now been corrected (S1 Appendix, pg. 2, line 39).

We thank you for this opportunity to resubmit to *PLOS ONE*, and we hope that our updated manuscript is suitable for publication in your journal.

Sincerely yours,

K. James Soda
Assistant Professor, Department of Mathematics and Statistics
Quinnipiac University
Email: Kenneth.Soda@quinnipiac.edu
Phone: 1(203) 582-7518
